# From precision medicine to cancer care through the immunome: highlights from the European Society of Medical Oncology Congress, Madrid, 26–30th September 2014

**DOI:** 10.3332/ecancer.2014.472

**Published:** 2014-10-16

**Authors:** Giuseppe Curigliano

**Affiliations:** Division of Experimental Therapy, European Institute of Oncology, Via Ripamonti 435, Milan 20141, Italy

**Keywords:** ESMO 2014, conference report

## Abstract

The recognition that cancer is a ‘spectrum’ of diseases, and that medical oncologists should achieve ‘convergence’ from ‘divergence’ to treat cancer patients was the main theme of the 2014 European Society of Medical Oncology (ESMO) Congress. The meeting assembled 19,859 participants from nearly 134 countries worldwide. The educational content was centered on precision medicine in cancer care, from mutational burden to the immunome, through the epigenome and the proteome. Precision medicine has been defined as the tailoring of medical treatment to the characteristics of an individual patient. Knowing an individual’s genomics has created a remarkable and unprecedented opportunity to improve medical treatment and develop preventative strategies to preserve health. Clinical oncologists across the range of diseases recognise that for precision medicine to take hold, it will require intensive, rigorous validation that these new approaches do indeed improve patient outcomes. Not all molecular alterations are predictive of response to a specific targeted treatment nor are they all druggable, raising issues of cost–benefit, validation of specific biomarkers, and of managing patient expectations. Addressing all these issues will be essential for the medical community to embrace any given opportunities. Along with it, it will also require educational programmes that squarely address the knowledge chasm that currently exists for practicing physicians. The promise of genomic and precision medicine has created greater demands for both those providing the scientific expertise—bioinformatics, statisticians, molecular biologists—and those delivering clinical care—physicians, nurses, psychologists—to the patients. This ESMO 2014 report will highlight the major findings of this outstanding meeting.

## Introduction

The availability of next-generation human genomic sequencing tools and the progress in sequencing and bio-computational technologies will enable genome-wide investigation of somatic mutations in human cancers at diagnosis and during their natural history [1, 2]. The theme for European Society of Medical Oncology (ESMO) Congress 2014 was ‘Precision Medicine in Cancer Care’. Genomic sequencing studies focuses on the comparison between the sequences found in tumour samples and those of the originating normal tissues, or those in the metastatic site of disease. The goal of this comparison is to identify regions of the genome that differ frequently enough to warrant further investigation of potential causal mechanisms. These studies have the potential to highlight underlying mechanisms of metastasis and resistance to drugs. Any cancer arises as a result of clonal expansions driven by cells that acquire a selective survival advantage through specific mutations. Genome-wide sequencing studies will therefore identify two specific types of mutations: the ‘drivers’—those providing a survival and proliferation selective advantage—and the ‘passengers’—those neutral to the selection process [3]. One of the major goals of the analysis of data from genome-wide sequencing studies is the ranking of genes based on the likelihood that they may be drivers. This is a new way to represent the ‘wiring diagram’ of cancer, identifying all molecular pathways that emphasises the heterogeneity and complexity of human cancer, explains mechanisms sustaining proliferation hallmarks of cancer and ‘drive’ tumour progression, and resistance to chemotherapy and targeted agents. The identification of druggable targets within these pathways represents a challenging platform for new drugs discoveries in patients with breast cancer [4]. Molecular characterisation of cancer subpopulation and molecular screening tools allowed the discovery of multiple oncogenic molecular alterations. A large number of such oncogenic events occur in a small percentage of cancer patients and define a specific segment of the disease. Disease segmentation in rare molecular entities is also related to a combination of frequent events [5]. The identification of such molecular events may be crucial in understanding molecular mechanisms inducing resistance to first-line therapy. Molecular screening of pathways upregulated in resistant tumours will have a major implication in early drug development. This conference report will highlight the major findings in solid tumours presented during the meeting.

## Breast cancer

The Presidential Symposium 1 was opened with data from the CLEOPATRA trial (pertuzumab, trastuzumab, and docetaxel for HER2-positive metastatic breast cancer trial). Prof. Sandra Swain of the Medstar Washington Hospital Centre, Washington Cancer Institute, reported that first-line treatment with pertuzumab/trastuzumab/docetaxel significantly improved overall survival (OS) for patients with HER2-positive metastatic breast cancer compared with placebo/trastuzumab/docetaxel, providing a 15.7 month increase in the median values. The median OS for the dual targeting arm was 56.5 months in first-line, and this substantial improvement confirms pertuzumab-containing regimens as the standard of care in this setting. In this trial, 808 patients with HER2-positive metastatic breast cancer were randomised to receive first-line placebo/trastuzumab/docetaxel or pertuzumab/trastuzumab/docetaxel. The patients were eligible for the study if they had HER2-positive (centrally confirmed), metastatic, locally recurrent, or unresectable breast cancer, measurable or non-measurable disease; had received ≤ 1 hormonal regimen for metastatic breast cancer prior to randomisation, disease-free interval of at least 12 months since prior neoadjuvant treatment, and left ventricular ejection fraction (LVEF) ≥ 50% at baseline. The study’s primary endpoint was progression-free survival (PFS), was independently assessed. Secondary endpoints included investigator-assessed PFS, objective response rate, safety, and OS. Final analysis was planned at 385 deaths, with two interim analyses at 165 and 267 deaths. At median follow-up of 50 months (range: 0–70 months), a statistically significant improvement in OS in favour of pertuzumab/trastuzumab/docetaxel arm was observed [hazard ratio (HR) = 0.68, *p* = 0.0002]. Median OS was 40.8 months in the placebo arm and 56.5 months in the pertuzumab arm, with a difference of 15.7 months. The OS benefit in predefined subgroups was consistent with previous observations [6]. It is to be noted that following the previous report of OS benefit, 48 patients in the placebo arm crossed over to the pertuzumab arm. The PFS in pertuzumab arm was 18.7 versus 12.4 months in placebo arm, HR 0.68 (*p* < 0.0001). Median time on study treatment was 17.4 months in pertuzumab arm versus 11.4 months in placebo group. The safety profile of pertuzumab and trastuzumab plus chemotherapy was consistent with the known safety profile of patients with long-term exposure to dual targeting. This means that we now have a treatment that improves OS and PFS without affecting the quality of life of patients in terms of cardiac safety. The potential of dual targeting in the adjuvant setting is being investigated in the ongoing APHINITY trial [Study of Pertuzumab in Addition to Chemotherapy and Herceptin (Trastuzumab) as Adjuvant Therapy in Patients with HER2-Positive Primary Breast Cancer], which is comparing the dual targeting of pertuzumab plus trastuzumab with trastuzumab alone following adjuvant chemotherapy. Dr Luca Gianni, from San Raffaele Hospital in Milan, Italy, discussed the study results. He highlighted that adjuvant trastuzumab was administered in only 10% of the study population. Dr Gianni said that the therapeutic role and wide applicability of dual HER2-blockade with monoclonal antibodies is established but newer therapeutic approaches to improve the overall results of CLEOPATRA to address the different biology and different drug sensitivity of subsets of HER2-positive tumours should be done. Improvements can be expected by addressing key features of HER2-positive breast cancer linked to different sensitivity in term of hormone receptor status (positive versus negative), PIK3CA status (wild type versus mutant), and immune environment. The CLEOPATRA study did not allow endocrine therapy of patients with ER-positive tumours. Dr Gianni wondered if the addition of endocrine therapy after the end of chemotherapy can increase the already large benefit observed in women with HER2-positive/ER-positive metastatic breast cancer patients enrolled in the CLEOPATRA study. The PIK3CA status can be easily assessed on tumour biopsies or liquid biopsies, and this molecular alteration may be predictive of response to dual targeting against HER2 receptor. Immune mechanisms and tumour lymphocyte infiltration are involved in the probability of pathologic complete response (pCR) in HER2-positive breast cancer. There is a high expression of programme death ligand (PDL)1 and cytotoic T-lymphocyte associated (CTLA)4 linked to residual disease in ER-negative tumours. Dr Gianni concluded that tests should be carried out to see if blocking of CTLA4 and/or programme death (PD)1/PDL1 will be useful for some patients treated per the CLEOPATRA protocol.

In the metastatic breast cancer session Joseph Gligorov, from Institut Universitaire de Cancerologie Université Pierre et Marie Curie, Paris, France, reported on the randomised phase III IMELDA trial (maintenance capecitabine and bevacizumab versus bevacizumab alone after initial first-line bevacizumab and docetaxel for patients with HER2-negative metastatic breast cancer) [7]. The trial addressed the role capecitabine added to maintenance bevacizumab after initial treatment with bevacizumab and docetaxel in this setting. In the present study 284 patients received initial bevacizumab and docetaxel; 185 (65%) were randomly assigned (91 to bevacizumab and capecitabine versus 94 to bevacizumab only). PFS was significantly longer in the bevacizumab and capecitabine group than in the bevacizumab only group (median 11.9 months [95% confidence interval (CI) 9.8–15.4] versus 4.3 months [3.9–6.8]; stratified hazard ratio 0.38 [95% CI 0.27–0.55]; two-sided log-rank *p* < 0.0001), as was OS (median 39.0 months [95% CI 32.3-not reached] versus 23.7 months [18.5–31.7]; stratified HR 0.43 [95% CI 0.26–0.69]; two-sided log-rank *p* = 0.0003). The most common grade 3 or worse events were hand-foot syndrome (28 [31%] in the bevacizumab and capecitabine group versus none in the bevacizumab alone group), hypertension (eight [9%] versus three [3%]), and proteinuria (three [3%] versus four [4%]). Serious adverse events were reported by ten (11%) patients in the bevacizumab and capecitabine group and seven (8%) patients in the bevacizumab only group. Despite the fact that data about post-progression treatment are lacking and endocrine treatment was not permitted in hormone-responsive population treated with bevacizumab, these results might inform future maintenance trials and current first-line treatment strategies for HER2-negative metastatic breast cancer. G. von Minckwitz from the German Breast Group reported on the randomised phase III TANIA study (bevacizumab plus chemotherapy versus chemotherapy alone as second-line treatment for patients with HER2-negative locally recurrent or metastatic breast cancer after first-line treatment with bevacizumab plus chemotherapy) [8]. The authors assessed the efficacy and safety of bevacizumab therapy beyond progression in patients with locally recurrent or metastatic breast cancer. Patients were randomly assigned [1:1] to receive second-line single-agent chemotherapy either alone or with bevacizumab. Second-line therapy was continued until the time of disease progression, unacceptable toxicity, or consent withdrawal. At progression, patients randomly assigned to chemotherapy alone, received third-line chemotherapy without bevacizumab; those randomly assigned to bevacizumab continued bevacizumab with third-line chemotherapy. The primary endpoint was PFS from randomisation to second-line progression, or death in the intention to treat population. In the study 494 patients were randomly assigned to treatment (247 in each group). PFS was significantly longer for those patients treated with bevacizumab plus chemotherapy than for those with chemotherapy alone (median: 6.3 months [95% CI 5.4–7.2] versus 4.2 months [3.9–4.7], respectively, stratified hazard ratio [HR] 0.75 [95% CI 0.61–0.93], two-sided stratified log-rank *p* = 0.0068). These results suggest that continued VEGF (vascular endothelial growth factor) inhibition with further bevacizumab is a valid treatment option for patients with locally recurrent or metastatic HER2-negative breast cancer whose disease was stabilised or responded to first-line bevacizumab with chemotherapy. Prof. Fabrice Andrè from Institute Goustave Roussy, Paris, reported on SAFIR01 and MOSCATO trial, 2 seminal European studies on precision medicine [9]. They stained 280 samples from metastases for immune analyses and for tumour infiltrating lymphocyte (TIL) assessment. Using a 50% cut-off, few tumours presented intratumoural (*n* = 3/244, 1%) and stromal (*n* = 11/244, 5%) TILs. This rate was significantly higher in HER2+ tumours (stromal TIL, 16%; *p* = 0.0002). Positivity for PD1 (*n* = 14/252, 5%) and PDL1 (*n* = 7/255, 3%) were rare, compared to reported in other tumour types. A trend towards higher PDL1 was observed in HER2+ mBC (8.3%; *p* = 0.0653). Ninety-three samples were analysed for whole exome sequencing (WES). When analysing genes specifically mutated in metastases, an enrichment in pathways involved in mitogen-activated protein kinase (MAPK) (false discovery rate (FDR) = 0.0035), estrogen receptor (ER) signalling (0.0004), lipids metabolism (0.0001), and gonadotropin releasing homone (GNRH) signalling (FDR = 0.00018) was observed. This is the first study that assesses both immune and genomic landscapes in male breast cancer (mBC). Authors reported that metastatic breast cancer dramatically differs from primary tumours, and is enriched in genes potentially involved in resistance mechanisms (ESR1, TSC1) or migration process (FRAS1, SCAPER). TILs, PD1, PDL1 are at very low frequency in metastatic lesions, except for Her2 + ++ mBC [9].

In ‘Breast Cancer, Early Stage’ session Dr Bonnefoi from Bordeaux, France reported on the EORTC 10054 study. In this trial, 128 patients with HER2-positive breast cancer received six cycles of chemotherapy every three weeks (three cycles of docetaxel 100 mg/m^2^ followed by three cycles of fluorouracil 500 mg/m^2^, epirubicin 100 mg/m^2^, cyclophosphamide 500 mg/m^2^). They were randomised to receive during the first three cycles either lapatinib (L) in arm A, trastuzumab (T) in arm B, or T and L in arm C. The primary endpoint was pathological complete response (pCR) rate defined as ypT0/is. The dual targeting increase pCR rate (56.3 versus 38.1%) [10]. Valentina Guarneri et al, Padova, Italy, reported on the correlation of PIK3CA mutational status with pCR in patients with HER2-positive early breast cancer (EBC) treated with neoadjuvant chemotherapy plus trastuzumab, lapatinib, or both [11]. In the whole population, pCR rates are similar in PIK3CA wild type (wt) and PIK3CA mutated patients (33.3% versus 22.7%; *p* = 0.34). However, for patients receiving T plus L (*n* = 41) the probability of achieving a pCR is higher in case of PIK3CA wt (48.5% versus 12.5%; *p* = 0.06). In this hypothesis-generating analysis, the increased activity of the dual anti-HER-2 blockade with T plus L seems limited to tumours not harbouring PIK3CA mutations.

## Genitourinary tumours and prostate cancer

Data from randomised phase III trials presented at the Proffered Paper Session on genitourinary and prostate showed a significant OS benefit in patients with ‘high-volume’ metastatic prostate cancer receiving combined antiandrogen and docetaxel as first-line treatment and have suggested the benefits of adding local radiotherapy to androgen deprivation in newly diagnosed high-risk non-metastatic (M0) prostate cancer [12]. High-volume prostate cancer is a poor prognostic factor in patients with hormone-naïve metastatic prostate cancer. A retrospective subanalysis from the randomised CHAARTED study in 790 men with first-line metastatic prostate cancer was reported by Dr Christopher Sweeney from the Dana Farber Cancer Institute, Boston, Massachusetts, USA. The addition of docetaxel to ADT in patients with high-volume disease improved OS from 32.2 to 49.2 months (*p* = 0.0013). Additionally, combined chemohormonal treatment among 518 men resulted in an improvement of prostate-specific antigen (PSA) < 0.2 at 12 months (*p* = 0.0011), time to PSA or clinical progression (*p* < 0.0001), and time to clinical progression (*p* < 0.0001). These results are of substantial clinical relevance and will change practice in patients with high-volume untreated metastatic prostate cancer. The investigators demonstrated that patients with ‘high-volume’, castration-sensitive metabolic disease benefit from upfront docetaxel, and it appears to confer a survival benefit that is superior to docetaxel given for metastatic castration-resistant disease. However, there is a need to develop better models to determine which ‘high-’ and ‘low-’ volume disease groups should be included to avoid, for example, discrimination between a patient with several small lesions and one with a single large lesion. Dr Charles Ryan from the University of California, San Francisco, USA, reported the final OS results of the randomised COU-AA-302 study in 1088 patients comparing the selective CYP17 inhibitor, abiraterone acetate, plus prednisone over prednisone alone in chemotherapy-naïve metastatic castration-resistant prostate cancer (CRPC) [13]. Disease progression and survival benefits had previously been reported in a planned interim analysis. With a median follow-up of 49.4 months, abiraterone acetate plus prednisone significantly prolonged OS compared with prednisone alone (median OS 34.7 months versus 30.3 months; hazard ratio [HR] 0.80; 95% confidence intervals [CI] 0.69–0.93; *p* = 0.0027). In future trials it will be important to select patients responsive versus resistant to abiraterone or other anti-androgen agents. It has recently been shown that the detection of androgen-receptor splice variant 7 messenger RNA (AR-V7) in circulating tumour cells from men with advanced prostate cancer was associated with resistance to enzalutamide and abiraterone. Among men receiving enzalutamide, AR-V7-positive patients had lower prostate specific antigen (PSA) response rates than AR-V7-negative patients (0% versus 53%) and shorter PSA PFS (median 1.4 months versus 6.0 months; *p* < 0.001) and OS (median, 5.5 months versus not reached; *p* = 0.002). Similar findings were made among men receiving abiraterone. These findings require large scale prospective validation [14]. This OS benefit was maintained despite the fact that 44% of patients in the control arm subsequently received abiraterone acetate plus prednisone. Tolerability was acceptable, although grade 3–4 adverse events of special interest were more common in the combination arm. These results confirm that the survival benefit of abiraterone acetate seen in patients who previously received chemotherapy is also apparent in chemotherapy-naïve metastatic CRPC [13]. Mc Dermott DF reported on the role of anti-PDL1 monoclonal antibody in metastatic clear cell renal carcinoma (mRCC). Among 69 mRCC patients evaluable for safety, 87% received prior systemic therapy including cytokines (39%), VEGF-inhibition (64%), and mTOR inhibitors (26%). Clinical activity was evaluated in 58 patients with clear cell histology; 51 patients (88%) had an evaluable baseline PD-L1 immunohistochemical status. The objective response rate (ORR) was 14% (8/58 partial responses (PRs), 95% CI: 6, 25) and the median duration of response was 54 weeks (2.7+ to 68.1+ weeks). The 24-week PFS rate was 53% (95% CI: 40.66). An association was seen between PD-L1 intensity and response to MPDL3280A.

## Gastrointestinal tumours

Dr Sargent reported on the CALGB/SWOG trial 80405. Patients with KRAS wt (codons 12 and 13) metastatic colorectal cancer received FOLFIRI or mFOLFOX6 and randomised to either cetuximab or bevacizumab. Accrual goal was 1142 patients. The endpoint was OS [16]. Investigators enrolled 3058 unselected patients, 2334 KRAS wt patients were randomised. In expanded RAS wt population, the median OS was pushed beyond 30 months. However, there was no significant difference between the cetuximab and bevacizumab in combination with chemotherapy (32 months versus 31.2 months). There was no difference in the PFS. However, there was higher response achieved in the cetuximab arm in the expanded RAS population, 68.6% versus 53.6% (*p* < 0.01) [16]. Dr R.S. Midgley from University of Oxford, UK, reported on the QUASAR2 trial. The aims of QUASAR2 were to assess whether the addition of bevacizumab to single agent capecitabine increases disease-free survival (DFS) and OS in colorectal (CRC) patients after resection of the primary; and to validate suggested, or discover new biomarkers of bevacizumab efficacy and toxicity. 1941 patients were randomised in a 1:1 ratio and demographics and disease characteristics were well balanced between the two arms. DFS in the whole trial population demonstrates that bevacizumab does not improve outcome in this setting (three year DFS 75.2% for CAP-BEV versus 78.2% for CAP: HR = 1.06; *p* = 0.54). Similarly OS was not improved (three-year OS 85.5% for CAP-BEV versus 87.2% for CAP; *p* = 0.38; HR = 1.12). There may be a temporal trend in HRs (HRs: one year 0.83 [0.61–1.13], two year 0.87 [0.65–1.17], three-year 1.32 [0.9–1.98]. Biomarker analyses confirm that high tumour stromal content confers a worse prognosis (three year DFS, HR 1.58 [1.22–2.05]; *p* = 0.001). Q2 supports data from two other trials suggesting no role for BEV in the adjuvant setting of CRC. In the non-colorectal session, data were presented on pembrolizumab in advanced gastric cancer [18]. Pembroluzimab is a highly selective, humanised IgG4/kappa isotype monoclonal antibody designed to block PD-1 interaction with its ligands PD-L1 and PD-L2, thus reactivating the immune system to eradicate the host tumour. In a phase IB study, authors assessed the safety, tolerability, and antitumour activity of pembrolizumab in gastric cancer patients. Using a prototype immunohistochemistry assay, PD-L1 expression was assessed in archival tumour samples from patients with recurrent/metastatic adenocarcinoma of the stomach or gastroesophageal junction. Of the 162 patients screened, 65 (40%) were PD-L1+, and 39 were enrolled. Median follow-up duration was six months. The most common adverse events (AEs) deemed treatment related by investigators were hypothyroidism and fatigue. Grade ≥ 3 AEs deemed treatment related occurred in three patients (*n* = 1 each for hypoxia, peripheral neuropathy, and pneumonitis). ORR (confirmed + unconfirmed) was 32% in Asia Pacific patients and 30% in the rest of the world. Evidence of an association between PD-L1 expression and PFS (P = 0.032) and ORR (P = 0.071) was observed.

## Gynaecological cancer

The final analysis of the NRG Oncology–Gynecologic Oncology Group Study on the role of bevacizumab in cervical cancer was reported. This phase III randomised trial was conducted using a 2 x 2 factorial design to determine whether chemotherapy plus bevacizumab and/or the non-platinum chemotherapy doublet (topotecan plus paclitaxel) improves OS in women with recurrent/persistent and metastatic cervical cancer. The primary endpoints were OS and toxicity with secondary endpoints being PFS and response. The final results of the study confirmed that the regimens administering bevacizumab continued to demonstrate a significant improvement in OS over chemotherapy alone: 16.8 months versus 13.3 months; HR 0.765 (95% CI: 0.62, 0.95; *p* = 0.0068). The benefit conferred by the incorporation of bevacizumab is sustained beyond 50 months as evidenced by the survival curves remaining separated. Data on rucaparib in BRCA-mutated patients have been reported [20]. Rucaparib is a potent, oral poly ADP ribose polymerase (PARP) inhibitor that induces synthetic lethality in homologous recombination deficient (HRD) tumours. Multiple mechanisms lead to HRD, which in turn leads to extensive genomic loss of heterozygosity (LOH). Patients (pts) with a BRCA mutation and/or high LOH may benefit from rucaparib treatment. Phase 1 enrolled 56 patients with advanced solid tumours (BRCA mutation and measurable disease not required) and established 600 mg b.i.d. as the RP2D. RECIST and/or CA-125 responses (two complete response (CRs), seven PRs, three CA-125) occurred in patients with ovarian, breast, or pancreatic cancer and a gBRCA mutation. At doses ≥ 360 mg b.i.d., disease control (CR + PR + SD > 24 weeks) in gBRCAmut OC patients was 82% (9/11), with 100% (3/3) of platinum-sensitive patients and 75% (6/8) of platinum-resistant patients deriving benefit. At the RP2D, 80% of gBRCAmut OC (3/4) and BC (1/1) patients had a RECIST or CA125 response. In addition, an OC patient, BRCA wt with high LOH in tumour, derived durable benefit (PFS = 36 weeks). Overall, the most common AEs were mild to moderate gastrointestinal (GI) effects and fatigue. At rucaparib doses ≥ 360 mg b.i.d., treatment-related AEs in ≥20% of patients (*n* = 27) (%G1/G2/G3) included nausea (33/15/4), fatigue (19/22/0), vomiting (26/11/0). Grade 3 lab abnormalities included low Hgb (*n* = 5, 17%), low platelets (*n* = 2, 7%), low ANC (*n* = 1, 3%), and increased ALT (*n* = 1, 3%). No grade 4 AEs occurred and no patient discontinued rucaparib because of an AE [20].

## Melanoma

Dr Eggermont from Paris, France, presented final data from the EORTC study 18071 [21]. In this randomised, double-blind trial, eligible patients included those ≥18 years of age who underwent complete resection of stage III cutaneous melanoma (excluding lymph node metastasis ≤1 mm or in-transit metastasis). 951 patients were randomised 1:1 to Ipilimumab 10 mg/kg (*n* = 475) or placebo (Pbo, *n* = 476) every three weeks for four doses, then every three months for up to three years until completion, disease recurrence, or unacceptable toxicity. The primary endpoint was recurrence-free survival (RFS). Secondary endpoints included safety and health-related quality of life (QoL). Overall, 20%/44%/36% of patients had stage IIIA/IIIB/IIIC, 42% ulcerated primary, and 58% macroscopic lymph node involvement. At a median follow-up of 2.7 years, Ipi significantly improved RFS versus Pbo (234/475 versus 294/476 events): median RFS 26.1 months for Ipi versus 17.1 months for Pbo (HR 0.75, 0.64-0.90; log-rank P = 0.0013). Three years RFS rates were 46.5% and 34.8%, respectively. RFS benefit was consistent across subgroups (e.g., stage IIIB or IIIC, ulcerated primary). Most common grade 3/4 immune-related adverse events (irAEs) in the Ipi and Pbo arms were gastrointestinal (15.9% versus 0.8%), hepatic (10.6% versus 0.2%), and endocrine (8.5% versus 0%). Most irAEs were managed and resolved using established algorithms. Of 471 patients who started Ipi, 245 (52%) discontinued treatment because of AEs (182 [38.6%] within 12 weeks); five (1.1%) died because of drug-related AEs. In this phase III trial, Ipi as adjuvant therapy provided a clinically and statistically significant improvement in RFS versus Pbo for patients with stage III melanoma at high risk of recurrence. AE profile was generally consistent with that observed in advanced melanoma, although with a higher incidence of e ndocrinopathies. Data from the COMBI-V trial have been reported [22]. First-line treatment with combination therapy dabrafenib plus trametinib improves OS in comparison with vemurafenib in patients with BRAF V600E/K mutation-positive unresectable or metastatic cutaneous melanoma. The results of a randomised, open-label, phase III, COMBI-v study were presented by Dr Caroline Robert of the Institut Gustave Roussy, Villejuif, France at Presidential Symposium 2. Combined BRAF and MEK inhibition, as compared with BRAF inhibition alone, delays the emergence of resistance and reduces toxic effects in patients who have melanoma with BRAF V600E or V600K mutations [22]. In this phase III trial, authors randomly assigned 423 previously untreated patients who had unresectable stage IIIC or stage IV melanoma with a BRAF V600E or V600K mutation to receive a combination of dabrafenib (150 mg orally twice daily) and trametinib (2 mg orally once daily) or dabrafenib and placebo. The primary endpoint was PFS. Secondary endpoints included OS, response rate, response duration, and safety. A preplanned interim OS analysis was conducted. The median PFS was 9.3 months in the dabrafenib–trametinib group and 8.8 months in the dabrafenib only group (hazard ratio for progression or death in the dabrafenib–trametinib group, 0.75; 95% confidence interval [CI], 0.57 to 0.99; P = 0.03). The overall response rate was 67% in the dabrafenib–trametinib group and 51% in the dabrafenib–only group (P = 0.002). At six months, the interim OS rate was 93% with dabrafenib–trametinib and 85% with dabrafenib alone (hazard ratio for death, 0.63; 95% CI, 0.42 to 0.94; P = 0.02). However, a specified efficacy-stopping boundary (two-sided P = 0.00028) was not crossed. Rates of adverse events were similar in the two groups, although more dose modifications occurred in the dabrafenib–trametinib group. The rate of cutaneous squamous-cell carcinoma was lower in the dabrafenib–trametinib group than in the dabrafenib-only group (2% versus. 9%), whereas pyrexia occurred in more patients (51% versus. 28%) and was more often severe (grade 3, 6% versus 2%) in the dabrafenib–trametinib group. Dr Robert concluded that a combination of dabrafenib and trametinib, as compared with dabrafenib alone, improved the rate of PFS in previously untreated patients who had metastatic melanoma with BRAF V600E or V600K mutations. coBRIM, a phase III, double-blind, placebo-controlled study of vemurafenib versus vemurafenib plus cobimetinib in previously untreated patients with BRAFV600 mutation-positive unresectable locally-advanced or metastatic melanoma was also presented [23]. Cobimetinib in combination with vemurafenib significantly improved PFS among patients with BRAFV600-mutant tumours. The results were reported by Prof. Grant McArthur of the Cancer Therapeutics, Peter MacCallum Cancer Centre, Melbourne, Australia in the Presidential Symposium 2. The combined inhibition of BRAF and MEK is hypothesised to improve clinical outcomes in patients with melanoma by preventing or delaying the onset of resistance observed with BRAF inhibitors alone. This randomised phase III study evaluated the combination of the BRAF inhibitor vemurafenib and the MEK inhibitor cobimetinib [23]. Authors randomly assigned 495 patients with previously untreated unresectable locally advanced or metastatic BRAF V600 mutation-positive melanoma to receive vemurafenib and cobimetinib (combination group) or vemurafenib and placebo (control group). The primary endpoint was investigator-assessed PFS. The median PFS was 9.9 months in the combination group and 6.2 months in the control group (hazard ratio for death or disease progression was 0.51; 95% confidence interval [CI] 0.39–0.68; P < 0.001). The rate of complete or partial response in the combination group was 68%, as compared with 45% in the control group (P < 0.001), including rates of complete response of 10% in the combination group and 4% in the control group. PFS as assessed by independent review was similar to investigator-assessed PFS. Interim analyses of OS showed nine month survival rates of 81% (95% CI, 75–87) in the combination group, and 73% (95% CI, 65–80) in the control group. Vemurafenib and cobimetinib was associated with a non-significantly higher incidence of adverse events of grade 3 or higher, as compared with vemurafenib and placebo (65% versus. 59%), and there was no significant difference in the rate of study drug discontinuation. The number of secondary cutaneous cancers decreased with the combination therapy [23]. Vemurafenib/cobimetinib combination, compared with vemurafenib alone, was associated with a higher incidence of grade ≥ 3 adverse events (65% versus 59%). However, there was no difference in the rate of adverse events leading to study drug discontinuation. Prof. McArthur concluded that the coBRIM study provides clear and definitive evidence that combined BRAF and MEK inhibition results in improved clinical outcomes. The addition of cobimetinib to vemurafenib was associated with a significant improvement in PFS among patients with BRAF V600-mutated metastatic melanoma, at the cost of some increase in toxicity [23].

## Lung cancer

Results of the IMPRESS trial have been reported by Dr T. Mock, from the Chinese University of Hong Kong, Hong Kong, China. Most patients with epidermal growth factor receptor (EGFR) mutation-positive non-small cell lung cancer (NSCLC) respond to first-line EGFR tyrosine kinase inhibitors, but later acquire resistance. The Phase III, double-blind IRESSA Mutation Positive Multicentre Treatment Beyond Progression Study (IMPRESS; NCT01544179) evaluated the efficacy/safety of continuing gefitinib plus cisplatin/ pemetrexed (cis/pem) (G) versus placebo plus cis/pem (P) in patients with acquired resistance to first-line gefitinib [24]. Patients (age ≥ 18 years [Japan ≥ 20 years], chemotherapy-naïve, locally advanced/metastatic NSCLC with an activating EGFR mutation, prior disease progression on first-line gefitinib) from 71 centres (Europe/Asia Pacific) were randomised to G or P (gefitinib 250 mg/day or placebo; plus cis 75 mg/m^2^/pem 500 mg/m^2^). Primary endpoint was PFS. Secondary endpoints included: OS, objective response rate (ORR), disease control rate (DCR), and safety/tolerability. Investigators randomised 265 patients (G = 133; P = 132). There was no statistically significant improvement in PFS for G versus P (hazard ratio [HR] 0.86; 95% confidence interval [CI] 0.65–1.13, *p* = 0.273; median PFS 5.4 months each). OS was immature (33% of patients had died), with better OS for P versus G suggested (statistically significant difference: HR 1.62; CI 1.05–2.52, *p* = 0.029). No treatment differences were found in ORR/DCR. Most common adverse events (AEs) in the safety population (G/P both *n* = 132): nausea (64%/61%), and decreased appetite (49%/34%); no interstitial lung disease noted. G was associated with increased grade 1/2 gastrointestinal toxicities. IMPRESS is the first and only randomised Phase III study to confirm continuation of gefitinib, in addition cis/pem would be of no clinical benefit for patients with acquired resistance to gefitinib; thus the standard of care should remain doublet chemotherapy alone. The safety profile for gefitinib plus cis/pem was in line with that known [24].

Of interest are the data from the MAGRIT trial [25]. This Phase III trial investigated whether the recMAGEA + AS15 cancer immunotherapeutic (MAGE-A3 CI) as adjuvant therapy improved DFS in patients with resected NSCLC. MAGRIT was a randomised, double-blind, placebo-controlled trial in patients with completely resected MAGE-A3-positive NSCLC Stages IB, II, and IIIA (TNM version 6) and who did or did not receive adjuvant chemotherapy. Patients were randomly assigned (2:1) to receive 13 intramuscular injections of MAGE-A3 CI or placebo over a 27-month (m) treatment period. The three co-primary endpoints were DFS in the overall and in the neoadjuvant chemotherapy population and DFS in patients with a potentially predictive gene signature. Out of 13,849 patients screened, 4210 patients had a MAGE-A3 positive tumour sample and 2272 patients were randomised and treated. Overall, 52% of the patients received adjuvant chemotherapy; 47%, 36%, and 17% were stage IB, II, and IIIA respectively. Median age was 63 years and 24% of patients were females. Mean relative dose intensity was above 98% in both groups throughout the treatment period. Median follow-up at the time of final analysis was 38.8m. Median DFS was 60.5m and 57.9m respectively for MAGE-A3 CI and placebo (HR 1.024, 95% CI 0.891–1.177; *p* = 0.7379). In patients who did not receive adjuvant chemotherapy, median DFS was 58.0m and 56.9m for MAGE-A3 CI and placebo groups, respectively (HR 0.970, 95% CI 0.797–1.179; *p* = 0.7572). The rate of grade ≥ 3 adverse events (16%) did not differ between treatment groups. Treatment of NSCLC patients with MAGE-A3 CI did not increase DFS compared to placebo in either the overall population or in patients who did not receive adjuvant chemotherapy. Because of the absence of treatment effect, a gene signature predictive of clinical benefit to MAGE-A3 CI could not be identified [25].

## Precision medicine in supportive care

ESMO 2014 also provided outstanding data on precision medicine in supportive care. The results from ROMANA 1 and 2 studies were reported by Dr D. Currow, Adelaide, Australia. ROMANA 1 and two trials were two international, double-blind, Phase III trials assessing anamorelin efficacy and safety in patients with unresectable stage III/IV NSCLC, Eastern Cooperative Oncology Group (ECOG) 0–2 and cachexia (≥5% weight loss within prior six months or BMI < 20 kg/m^2^) [26]. Cancer anorexia–cachexia syndrome is a common debilitating condition, characterised by decreased body weight, mainly lean body mass (LBM) and negatively impacts quality of life and prognosis. Anamorelin HCl (ANAM) is a novel selective ghrelin receptor agonist with appetite-enhancing and anabolic activity. Patients were randomised (2:1) to 100 mg ANAM or placebo, given daily orally for 12 weeks and were permitted to receive chemotherapy while on study. Co-primary endpoints were changed from baseline over 12 weeks in LBM (measured by DXA) and in handgrip strength (HGS). Secondary endpoints included change in body weight and in the anorexia–cachexia subdomain of the Functional Assessment of Anorexia/Cachexia Therapy (FAACT) questionnaire. Safety assessments included lab values and adverse events (AEs). There were no within-study population differences for ROMANA 1 (N = 484) and ROMANA 2 (N = 495). Over 12 weeks, ANAM significantly increased LBM versus placebo (*p* < 0.0001) in both studies. In ROMANA 1, median change in LBM was 1.10 kg [95% CI 0.76; 1.42] for ANAM versus-0.44 kg [95% CI–0.88; 0.20] for placebo; similarly, changes in ROMANA 2 were ANAM 0.75 kg (95% CI 0.51; 1.00) versus placebo-0.96 kg (95% CI–1.27; -0.46). Change in human genome science (HGS) was not statistically different between study arms. ANAM increased body weight (2.20 ± 0.3 versus 0.14 ± 0.4 kg; *p* < 0.0001; and 0.95 ± 0.4 versus-0.57 ± 0.4 kg; *p* < 0.0001) and improved FAACT subdomain scores (4.12 ± 0.8 versus 1.92 ± 0.8; *p* = 0.0004; and 3.48 ± 0.9 versus 1.34 ± 1.0; *p* = 0.0016). In the ANAM arm, most frequent drug-related AEs were hyperglycemia (5.3%) and nausea (3.8%) for ROMANA 1, hyperglycemia (4.2%) and diabetes (2.1%) for ROMANA 2. Both studies had few drug-related grade ≥ 3 AEs (0.9%, 2.7%). In two global, large-scale Phase III studies, anamorelin for 12 weeks was well tolerated, and significantly improved the LBM, body weight, and anorexia–cachexia symptoms/concerns in advanced NSCLC patients with cachexia.

## Conclusions

There are numerous challenges that need to be overcome to successfully implement personalised cancer therapy. These include biological challenges such as tumour heterogeneity and molecular evolution, technical challenges such as limitations of molecular tests, pharmacological challenges such lack of effective drugs, and regulatory and reimbursement challenges [27, 28]. 1) Tumour heterogeneity. During tumour progression, subclones frequently arise, which results in differences in the proportion and pattern of specific aberrations between the primary tumour and the metastases or recurrent tumours that originate from it. Strikingly, metastases are not necessarily more complex than the primary tumour from which they originated, and can actually lose aberrations that are present in the primary lesion. There may also be significant intratumoural heterogeneity, with spatially separated heterogeneous somatic mutations and chromosomal imbalances. 2) Molecular evolution and resistance. The array of clones with particular aberrations can change under both the selective pressure of a targeted therapy and as a result of the mutagenic activity of radiation and chemotherapy. There are two general conceptual approaches to deal with intratumoural heterogeneity and emergence of resistance: in-depth characterisation of tumours and recurrence to identify rare and dominant clones, and low-depth sequential characterisation of tumours to identify dominant clones. Repeated biopsies at progression can assist in determining whether emergent aberrations are mediating resistance and whether these aberrations could be therapeutically treated. As obtaining multiple biopsies is costly and associated with potential morbidity, surrogates such as molecular imaging, or analysis of circulating tumour cells or circulating free DNA, are also being pursued in ongoing studies. 3) Undruggable targets. The role of in-depth molecular analysis is to identify molecular aberrations that can be targeted with existing therapeutic strategies. However, many proteins are currently ‘undruggable’, and loss-of-function mutations in many tumour suppressor genes, such as TP53, are currently not actionable. However, our drug toolkit is rapidly evolving and emerging technologies that interrupt protein–protein and DNA–protein interactions, and approaches such as siRNA can potentially make many previously undruggable targets druggable. With the development of RNA interference libraries, systematic and cost-effective genome-wide loss-of-function screenings can now be carried out to assess the role of specific genes in tumourigenic phenotypes, and to identify novel drug targets in cells with specific genomic aberrations. 4) Technical challenges. Identification and validation of markers of sensitivity and resistance is a key step in the implementation of personalised cancer therapy. In early clinical trials, generally carried out in heavily pretreated patients with advanced-stage or metastatic disease, patients occasionally demonstrate unexpected responses—including complete or partial responses—or unfortunately, in some cases, rapid disease progression. In-depth characterisation of these ‘unusual responders’ has identified important biomarkers of sensitivity. Personalised cancer therapy programmes [27, 28]. Comprehensive analysis of not only alterations in the genome but also the epigenome, transcriptome, proteome, and gene–gene, protein–protein and genome–environment interactions is likely to have important clinical implications in biomarker development. 5) Need for new trial designs. Novel clinical trial designs to identify and validate biomarkers and targeted therapeutics require special approaches such as mandatory research biopsies, and comprehensive tumour and germline characterisation. Personalised therapy trials may require tissue samples to be collected through a research biopsy before therapy to assess predictive markers, during therapy to assess pharmacodynamic markers of response, and at the time of tumour progression to assess mechanisms of therapy resistance. Biomarker discovery and validation must be integrated into all aspects of drug development, from discovery through to clinical trials. However, current clinical trial designs are often not optimal for biomarker discovery and validation. Owing to costs and potential delays in patient accrual, early clinical trials frequently do not incorporate molecular testing or biopsies, however, biomarkers are getting increasingly incorporated into trials assessing biological agents. 6) Pharmacological challenges. Although multiple targeted therapies are currently entering clinical trials, it is not yet clear whether these agents have the appropriate specificity, pharmacology, and pharmacodynamics to inhibit their therapeutic targets. There is a real risk of abandoning outstanding targets due to studies of therapeutic agents with poor or variable bioavailability, short half lives or off-target toxicity delivered with inappropriate dosing. Indeed, with very few exceptions, it is not yet known what degree and duration of target inhibition is necessary to develop optimal outcomes.

The ‘wiring diagrams’ of breast cancer subtypes define that the signalling circuitry describing the intercommunication between various pathways should be charted in far greater detail and clarity, in order to better understand ‘drivers’ and ‘passengers’. The number of potential driver genes is large, even if more limited is the number of ‘driver’ pathways. Patient selection, rational combination therapies, surrogate markers identification, and tumour tissue banking will be key areas of research. Research efforts should be directed at generating the level of evidence required to make comprehensive testing reimbursable. Until that time, partnerships between academia and industry, as well as significant philanthropic support are needed to facilitate comprehensive molecular characterisation to demonstrate that it benefits patients.
